# Effects of Vitamin D on Apoptosis and Quality of Sperm in Asthenozoospermia

**DOI:** 10.5935/1518-0557.20200009

**Published:** 2020

**Authors:** Mahin Taheri Moghadam, Ghazal Hosseini, Forouzan Absalan, Mahmoud Hashemi Tabar, Roshan Nikbakht

**Affiliations:** 1 Cellular and Molecular Research Center, Ahvaz Jundishapur University of Medical Sciences, Ahvaz, Iran; 2 Department of Anatomical Sciences, Faculty of Medicine, Ahvaz Jundishapur University of Medical Sciences,Ahvaz, Iran; 3 Fertility, Infertility and Perinatology Center, Imam Khomeini Hospital, Ahvaz Jundishapur University of Medical Sciences, Ahvaz, Iran; 4 Department of anatomical sciences, Abadan School of Medical Sciences, Abadan, Iran

**Keywords:** apoptosis, asthenozoospermia, sperm motility, sperm morphology, vitamin D

## Abstract

**Objective:**

Vitamin D receptor (VDR) is expressed in human spermatozoa. However, the role of vitamin D (VD) in human male reproduction has not yet been clarified. In this study, effects of VD on sperm parameters and its apoptosis in asthenozoospermic and healthy men were evaluated.

**Methods:**

The study was carried out on discharged semen samples of 80 asthenozoospermic and healthy men. The samples were divided into control and experimental groups (received 20 µMol of VD). This study assessed sperm motility using the Makler chamber, their morphology by Diff quick, apoptosis and necrosis by Annexin-V and TUNEL assays, and their chromatin integrity was assessed by Aniline blue and Toluidine blue staining, according to WHO guidelines.

**Results:**

The results revealed that: 1) the total number of motile sperms was increased by VD in both groups, but it was only significant in the asthenozoospermia group. 2) The progressive motility was increased with significant difference in both groups.3) Morphology of sperm did not show any changes due to VD in any of the groups. 4) Early apoptosis and necrosis of sperms were reduced in both groups, but the results of late apoptosis showed no statistical difference in these groups. 5) The percentage of positive toluidine blue was significantly decreased after using VD in the asthenozoospermia group.

**Conclusion:**

VD could improve motility, early apoptosis, and sperm necrosis, especially in asthenozoospermic men and it could be used for therapeutic opportunities.

## INTRODUCTION

Infertility is a common disorder with significant medical, psychosocial, and economic aspects (Benyamini *et al*., 2005), which has had a worldwide increase, and now approximately 1/6 of the couples are struggling with it (Thonneau *et al*., 1991). The male factor infertility remains a significant problem contributing to 50% of the cases visiting infertility clinics (Schulte *et al*., 2010). Asthenozoospermia is a common cause of male infertility, in which motile sperm is less than 40% and progressive motile sperm is less than 32% (WHO, 2010), which could even be 0% (absolute immotile) (Ortega *et al*., 2011). Sperm motility is extremely important for migration from the vagina to the fallopian tubes, penetration of the cumulus oophorus, and for processes involved in fertilization (Ortega *et al*., 2011); thus asthenozoospermia has a poor fertility prognosis (Beauchamp *et al*., 1984). Some studies showed that apoptosis could affect sperm motility and lead to poor sperm motility and asthenozoospermia (Moradian Fard *et al*., 2019).

Apoptosis is physiologically-programmed cell death, which is different from necrosis and affects cells without any related inflammation in the surrounding tissue (Wyllie *et al*., 1980). Some reports have demonstrated that ejaculated spermatozoa from infertile men show ultrastructural damage, an unusually high incidence of DNA fragmentation, and plasma membrane translocation of phosphatidylserine (PS), all of which are typically considered to be signs of apoptosis in somatic cells (Barroso *et al*., 2000).

Nonetheless, these sperms may be considered normal in routine semen analyses. Under certain conditions like ICSI, these sperms could carry a damaged genome into the oocyte, resulting in serious consequences (Bedu-Addo *et al*., 2008). Today, in addition to assisted reproductive techniques (ART), researchers are considering the development of new techniques to increase sperm motility and improve apoptosis, moving towards a better management of the infertile patients (Moradian Fard *et al*., 2019).

There is evidence that Vitamin D (VD) modulates reproductive processes in women and men (Luk *et al*., 2012). VD deficiency and Vitamin D receptor (VDR) mutation in rodents have caused uterus hypoplasia, damaged follicles in the female reproductive system and decreased sperm count and motility with histological abnormalities of the testis in male rats (Kinuta *et al*., 2000; Aquila *et al*., 2009). Insufficient VD causes increased frequencies of chromosomal aberrations and sister chromatid exchanges due to oxidative, hypoxic, and apoptotic stresses (Benyamini *et al*., 2005). In addition, it causes incomplete spermatogenesis and degenerative changes as well (Ramlau-Hansen *et al*., 2011). Some studies have shown that the serum and seminal plasma vitamin D may be involved in regulating sperm quality (morphology, concentration, motility and acrosome reaction) (Aquila *et al*., 2009; Ramlau-Hansen *et al*., 2011; Blomberg Jensen *et al*., 2011; Jueraitetibaike *et al*., 2019), and others showed that vitamin D has anti-apoptosis function in some cells (Zhang *et al*., 2007); therefore, the authors decided to assess the role of this vitamin in apoptosis and sperm quality in asthenozoospermic and healthy men. For this purpose, we used VD under *in vitro* condition like ART and evaluated sperm quality (morphology, motility, chromatin integrity) and apoptosis using Annexin V and TENNEL. Provided that positive effects are observed on these parameters, VD could be used in future infertility treatment and ART.

## MATERIAL AND METHODS

### Semen Collection and Processing

We ran this experimental study on discharged semen samples of 40 asthenozoospermic and 40 healthy men referred to an IVF infertility clinic. The Ethics Committee of the Research Deputy of Ahvaz Jundishapur University of Medical Sciences (IR.AJUMS.REC.1395.266) approved this research. Since vitamin D may have a positive effect on sperm parameters and can be used in future IUI and IVF methods, this project fulfills all the requirements for sperm preparation and patient selection such as IVF methods. We took the samples from men without infection or varicocele. Human semen was collected into a sterile container after a 3-5-day period of sexual abstinence (Barroso *et al*., 2006). In this study, we defined asthenozoospermia when a man’s sperm motility is less than 40% and he has less than 32% progressive motile sperm. In contrast, a healthy man is defined as a person who has the following parameters: sperm count >20×10^6^ ml, sperm motility >40%, normal morphology >4%, and leukocyte <1×10/ml (WHO, 2010). Samples were allowed to liquefy at 37°C for 30 min. 5µl of the liquefied samples were loaded on slides for determining normal and asthenozoospermic samples (Blomberg Jensen *et al*., 2011). Like IVF methods, semen samples were washed twice with 1ml fresh sperm wash (ALL Grad Wash, Life Global, 4264, Brussels, Belgium) and centrifuged at 1,800 rpm for 5 min. After removing the supernatant, 1 ml fresh sperm wash was added and the mixture was kept at a 45° angle in an incubator at 37°C for 45 min so that motile sperms could swim up. After that, the supernatant was divided into two parts; one part was considered as control group and the other one received 20µMol VD (based on the pilot work we did) and considered as experimental group. Both groups were incubated at 37°C for 1 hour (Blomberg Jensen *et al*., 2011).

### Evaluation of Basic Sperm Parameters

We evaluated sperm concentration, motility, and morphology of both the original raw samples and the experimental groups. We placed 5µl of each sample into a Makler chamber and the sperm was counted under light microscopy at 40× magnification to assess progressive sperm motility (%), total motility (%) and total sperm count per ejaculation (million sperm). We analyzed at least 200 spermatozoa per slide. We classified the sperm as progressive motile (class A+B), non-progressive motile (class C), or immotile (class D).

We evaluated sperm morphology according to strict criteria at 40×magnification (Barroso *et al*., 2006). Each sample (10µl) was spread along the slide and allowed to dry for 20 minutes before staining with Diff-Quick staining. We evaluated an average of 200 spermatozoa per slide, twice, by two examiners.

### Apoptosis detection

#### PS with Annexin-V Externalization Evaluation

In the early apoptotic events, PS was translocated from the internal membrane to the external membrane of cells. Annexin-V is a calcium-dependent phospholipidic union protein with a high affinity for PS. It can bind to PS and distinguish apoptotic cells. For this detection, according to the Annexin V protocol (Annexin-V-FLOUS Staining kit, No.11 858 777 001, Roche, Mannheim, Germany), 2 µl Annexin-V solution, 100 µl additional binding buffer, and 2 µl propidium iodide (PI) were added to the samples in a dark place and the mixture was left there for 20 minutes. After this step, the samples were checked under a fluorescence microscope. At least 200 cells per slide were analyzed in a randomized manner and they were identified as either normal (negative for Annexin-V and PI, pale stain), apoptotic (positive for Annexin-V, green stain, and negative for PI), or necrotic (positive for PI, red stain) cells (Barroso *et al*., 2006; Oosterhuis *et al*., 2000). 

#### DNA fragmentation detection

Apoptosis-related DNA strand breaks were evaluated by terminal deoxynucleotidyl transferase-mediated dUTP nick-end labelling (TUNEL), using the Apoptosis Detection System Florescence (in situ Cell Death Detection Kit, POD, No.11 684 817 910, Roche, Penzberg, Germany). We ran the procedure according to the manufacturer's instructions. We smeared the samples on slides, fixed with 100% methanol for 4 min, and incubated them in a blocking solution (3%H_2_O_2_ in methanol) for 20 min in a dark room. Then, the slides were rinsed with PBS and permeabilized with 0.1% Triton X-100 in PBS. The slides were washed twice in PBS and incubated in a TUNEL incubation buffer, (which contained nucleotide and the terminal transferase enzyme), at 37°C for 1 hour in a humidified chamber. After stopping the enzyme’s reaction, the slides were washed three times in PBS for 15 min. The slides were observed under a fluorescence microscope at 40× magnification. For each slide, 200 sperms were checked. Omitting Enzyme terminal transferase and D-Nase I were done for negative and positive controls. The slides were read twice and an average number was obtained. In these slides, cells with green fluorescence color were reported as TUNEL positive (Dominguez-Fandos *et al*., 2007).

#### Chromatin integrity detection

After obtaining the appropriate cells, 20 µl of the sample was prepared as smear. The smears were dried at room temperature; then, the fixation of each staining was added to the smears. With the aim of fixing the stain, the aniline blue fixative (3% glutaraldehyde) was added to the slides, at room temperature and they remained in place for 30 minutes. After that, the slides were stained with aniline blue solution (5% aqueous aniline blue solution with 4% acetic acid) for 10 minutes. In order to fix the toluidine blue staining smear, they were fixed under a refrigerated temperature by ethanol-acetone (1:1) for 30 minutes. After that, the slides were hydrolyzed by HCL 0.1% N for 5 minutes at 4°C. Subsequently, the samples were washed three times with distilled water and then toluidine blue solution (0.05% TB) was added to the samples and were kept for 10 minutes. After that, the cells were counted under an inverted light microscope. Cells with dark blue color were reported as abnormal and those with a light blue color were considered healthy. About 200 cells were counted in each slide.

### Statistical Analysis

Data analysis was carried out using the Statistical Package for the Social Sciences (SPSS version 22). The results were presented as mean ± SD and *p*-value. Normal distribution of the data was assessed by the Kolmogorov-Smirnov Z test. The data of the control group and the vitamin D-treated group were compared by the paired t-test with a significance level of *p*≤0.05.

## RESULTS

For determining normozoospermic and asthenozoospermic semen samples, we assessed sperm volume, concentration, total motility, and progressive motility, and the results are listed in Table 1.

**Table 1:** Mean of semen parameters from the normal (n=40) and asthenozoospermia samples (n=40)

Groups	Semen parameters	Mean±SD
**Normal**	Volume (mL)	1.66±1.25
	Sperm concentration (millions/ml)	99.20±30.90
	Total motility (%)	57.27±16.43
	Progressive motility (%)	41.42±16.45
	Immotile (%)	42.72±16.43
**Asthenozoospermia**	Volume (mL)	2.40±1.19
	Sperm concentration (millions/ml)	54.97±25.36
	Total motility (%)	17.70±10.49
	Progressive motility (%)	13.9±9.41
	Immotile (%)	82.45±10.53

### Conventional sperm parameters in study groups

#### Motility of sperm cells

Results of motility in the normal and asthenozoospermic groups showed that the percentage of total motile sperms was increased in the experimental group (receiving VD) in comparison with the control group. However, there was a significant difference in the asthenozoospermic group (*p*<0.0001). The progressive motility was increased by VD and the difference was significant in both groups (*p*=0.036 in the normal group and *p*<0.0001 in the asthenozoospermic group). Moreover, immotile sperms were decreased by VD in both groups, but this difference was significant (*p*<0.0001) in the asthenozoospermic group (Table 2).

**Table 2:** Mean of semen parameters from the normal (n=40) and asthenozoospermic (n=40) samples after incubation with vitamin D

Variables	Groups	Groups	Mean±SD	*p*-value
Concentration (millions/ml)	Normal	+VD	39.97±18.53	0.889
		Control	34.50±23.33	
	Asthenozoospermia	+VD	15.42±6.47	0.547
		Control	8.92±3.85	
Total motility (%)	Normal	+VD	33.67±17.41	0.088
		Control	24.47±20.18	
	Asthenozoospermia	+VD	10.1±6.43	<0.0001*
		Control	3.82±3.58	
Progressive motility (%)	Normal	+VD	27.05±17.65	0.036*
		Control	16.37±18.19	
	Asthenozoospermia	+VD	8.25±6.0	<0.0001*
		Control	1.9±3.41	
Immotile (%)	Normal	+VD	66.37±17.46	0.09
		Control	75.52±20.18	
	Asthenozoospermia	+VD	90.2±5.93	<0.0001*
		Control	96.17±3.58	

* *p*-value≤0.05

#### Sperm morphology

According to the results (Table 3), after using VD, no significant difference was observed in the percentage of sperms with normal morphology in the normal and asthenozoospermic groups.

**Table 3:** Mean of normal morphology of sperms in the normal (n=40) and asthenozoospermic samples (n.40) after incubation with vitamin D

Variable	Groups	Groups	Mean±SD	*p*-value
Morphology(%)	Normal	+VD	27.09±15.25	0.889
		Control	25.74±14.71	
	Asthenozoospermia	+VD	22.45±26.39	0.499
		Control	23.17±14.27	

### Detection of Apoptosis in the study groups

#### Apoptosis indicated by PS Expression (Annexin V assay)

**Figure 1:**
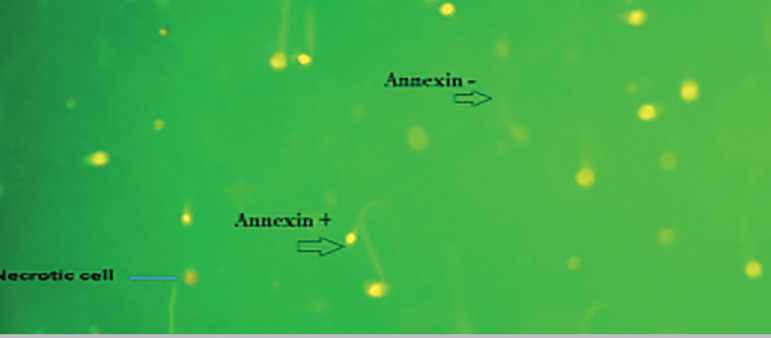
Annexin-V assay by the effect of VD on apoptosis in sperm. Annexin V staining+ (green color), Annexin V staining –(pale color) and necrotic cell (red color).

**Figure 2:**
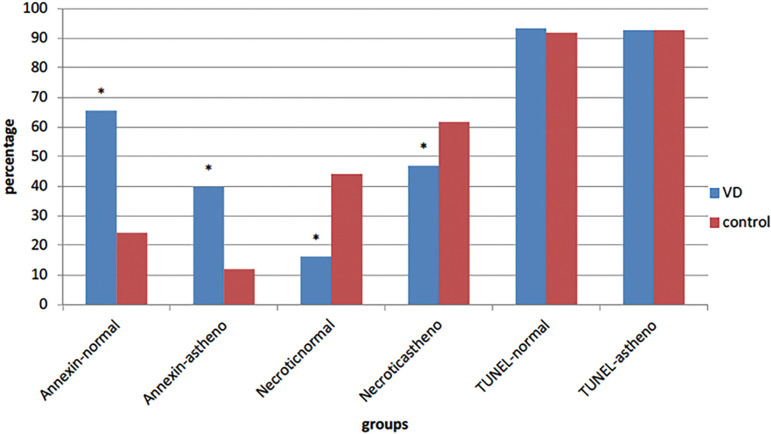
Apoptosis compression in experimental groups, *p-value<0.05

Annexin-V assay in normal and asthenozoospermic groups showed that adding VD causes an increase in the number of normal cells (pale green) in two groups and the mean apoptosis was decreased in these groups, with a significant difference (*p*<0.0001in both groups). The proportions of necrotic sperm (red color) (PI positive) was significantly different between the VD and control groups in normal (*p*=0.007) and asthenozoospermic individuals (*p*<0.0001) (Figs. 1 and 2).

#### Apoptosis Measured by DNA Double-Strand Breaks (TUNEL assay)

In this study, VD was not able to affect the percentage of DNA fragmentation, and data analysis showed no significant difference between the VD group and the control group in normal (*p* =0.446) and asthenozoospermic individuals (*p*=0.998) (Fig. 2).

### Detection of chromatin integrity

The chromatin integrity data showed that VD did not have a significant effect on the protamines of the sperms’ nuclei (aniline blue staining) in the normal and asthenozoospermic groups (*p*=0.799 and *p*=0.071, respectively); however, this vitamin significantly affected the phosphate groups (toluidine blue staining) of the sperms’ nuclei and caused an increase of normal cells in the asthenozoospermic group (*p*=0.033) (Table 4).

**Table 4:** Mean of chromatin integrity of the normal (n=40) and asthenozoospermic samples (n=40) after incubation with vitamin D

Variables	Groups	Groups	Mean±SD	*p*-value
AB- (%)	Normal	+VD	62.77±30.08	**0.799**
		Control	63.78±32.24	
	Asthenozoospermia	+VD	47.19±29.82	0.071
		Control	57.64±26.16	
TB-(%)	Normal	+VD	35.59±21.38	0.161
		Control	40.92±24.05	
	Asthenozoospermia	+V D	51.70±29.54	0.033*
		Control	39.05±23.86	

* *p*-value<0.05

## DISCUSSION

VD has been recognized for maintaining calcium, phosphorus homeostasis, and bone mineralization. There is some evidence that VD modulates the reproductive processes in men, and VDR and its metabolizing enzymes are expressed in human spermatozoa (Blomberg *et al*., 2011; 2011; Aquila *et al*., 2008). However, the role of this vitamin in human male reproduction has not been fully explained to this date (Aquila *et al*., 2009). In this study, we assessed the role of VD in the quality and apoptosis of sperms in healthy and asthenozoospermic men.

Regarding the effects of VD on sperm motility, this study showed that the total sperm motility was improved, and this improvement was significant in the asthenozoospermic group; moreover, progressive sperm motility was significantly increased in both groups. In addition, the immotile sperms were decreased with this vitamin in the asthenozoospermic samples. Therefore, VD could have an effect on sperm motility, especially in cases of asthenozoospermia. In agreement with this study, several studies have shown that VD serum levels are important for semen quality (Aquila *et al*., 2008; Blomberg *et al*., 2011). Blomberg Jensen *et al*. (2011) explained that VD serum levels are positively associated with sperm motility, and men with VD deficiency (<25 nM) had a lower proportion of total and progressive motile sperms compared to men with high VD levels (>75 nM). On the other hand, one study revealed that VDR knockout mice have much less motile sperms (Bouillon *et al*., 2008). Two other studies of fertile and infertile men showed that men with vitamin D sufficiency had more motile spermatozoa than those with vitamin D deficiency (Yang *et al*., 2012; Blomberg Jensen & Dissing, 2012), but another study reported that serum vitamin D levels had no relationship with semen parameter values in a fertile population, while in patients with oligoasthenozoospermia or teratozoospermia, there was a positive correlation between vitamin D and sperm quality (Abbasihormozi *et al*., 2017). By in vitro studies, Blomberg Jensen *et al*. (2011) and Aquila *et al*. (2009) showed that VD increases intracellular calcium concentration and sperm motility, and it induces acrosome reaction in mature spermatozoa from healthy men. Other studies showed that CYP24A1 (VD-inactivating enzyme) is co-expressed with VDR and it mediates a non-genomic increase in Ca^2+^ of the human sperm (Blomberg Jensen *et al*., 2012; Kong *et al*., 2007). In addition, one study explained that VD might enhance sperm motility by promoting the synthesis of ATP, both through the cAMP/PKA pathway and the increase in intracellular calcium ions (Jueraitetibaike *et al*., 2019). Although, Ca^2+^ was not assessed in our study, its increase may be responsible for the VD-mediated induction of sperm motility.

Moreover, in this study, normal sperm morphology was evaluated by Diff-Quick staining in both asthenozoospermic and normal groups, and no improvement in normal morphology of sperm was seen after adding the vitamin. With regards to the effects of VD on the morphology of sperms, Ramlau-Hansen *et al*. (2011) showed that high serum levels of VD was associated with low percentage of sperms with normal morphology; however, Blomberg Jensen *et al*. (2011) explained that men with VD deficiency (<25nM) had a low percentage of morphologically normal sperm, versus men with high VD levels (>75nM).This discrepancy with the current study’s data may be due to different methodological approaches. They evaluated the effects of VD in serum on sperm, but in this study, vitamin D was used under *in vitro* conditions, and as a result, VD was not effective concerning sperm morphology.

Concerning apoptosis in sperm, the fact is that it plays an important role in regulating spermatogenesis. Several features of apoptosis have been described in human sperm. These features include PS and DNA fragmentation. These markers are frequently found in the ejaculations of infertile men (Barroso *et al*., 2000) and today, researchers are looking for solutions to reduce apoptosis in the treatment of diseases (Zhang *et al*., 2007).

In this study, we investigated the effects of VD on apoptosis by PS externalization (Annexin-V assay) and DNA fragmentation (TUNEL assay) in sperm. The results revealed that VD caused a decrease in early apoptosis in the asthenozoospermic and normal groups, and the percentage of normal cells was significantly increased in the two groups. In explaining these results, one study showed that vitamin D could react with fatty acid residues in the cell membrane, by its hydrophobic parts and could protect it from disintegration (Wiseman, 1993). Moreover, in another study, the authors demonstrated that VD could improve the integrity of sperm membrane during freezing and thawing by decreasing ROS (Taheri Moghadam *et al*., 2019).

In this study as well, the proportion of necrotic sperm was decreased in both groups that were affected by vitamin D. However, the late apoptosis with TUNEL assay was not decreased in any of the groups, and this vitamin was not able to improve DNA fragmentation in sperm within one hour. Like these results, Koppers *et al*. (2011) induced apoptosis in spermatozoa by exposure to wortmanina and they showed caspase activation and PS externalization in sperm, but they did not see any TUNEL positivity. They explained that the spermatozoa has a mid-tail piece with the mitochondria and cytoplasm in a distinct subcellular compartment from the nucleus. As a result, even though endonucleases can be detected in apoptotic human spermatozoa they remain resolutely locked in the sperm mid-tail piece and never gain access to the nuclear compartment. In addition, Smith *et al*. (2013) showed that spermatozoa does not have APE1 that is needed to create the 3’-hydroxyl group, targeted by terminal transferases in the TUNEL reaction. Finally, sperm chromatin is so condensed and it is very difficult for the reagents used in the TUNEL assay to penetrate the chromatin and register the presence of a DNA strand break, and 48 hours after the apoptosis starts, the spermatozoa become TUNEL positive (Smith *et al*., 2013). Muratori *et al*. (2003) assessed the correlation between sperm’s DNA fragmentation and necrosis, and they stated that the sperms with early apoptosis may later die by necrosis, and DNA damage occurs further on during incubation. Another study showed that diets deficient in vitamin D could increase DNA fragmentation of animal sperms (Merino *et al*., 2018), and vitamin D has anti-apoptosis function in some cells (Zhang *et al*., 2007); therefore, in the current study, vitamin D may have improved the late apoptosis at other point-times during sample incubation (that lasted for more than an hour).

In addition to chromatin integrity detection, only toluidine blue staining was decreased in the asthenozoospermic group and VD was not able to completely improve the chromatin integrity of sperms. Sperm DNA is packed with protamines and the sperm nucleus does not allow for transcription or any other changes by other factors; thus VD, like late apoptosis, may be able to improve chromatin integrity in longer incubation periods.

## CONCLUSION

The present study revealed that VD could reduce early apoptosis as well as necrosis, and increase progressive motility in asthenozoospermic and healthy men; in addition, it improved total motility and immobility in asthenozoospermic men. Further studies are needed to determine the effects of VD on improving sperm function in asthenozoospermic men, which may have fundamental therapeutic implication in IVF and IUI assisting reproductive techniques.
